# The Brain of the Archerfish *Toxotes chatareus*: A Nissl-Based Neuroanatomical Atlas and Catecholaminergic/Cholinergic Systems

**DOI:** 10.3389/fnana.2016.00106

**Published:** 2016-11-11

**Authors:** Naomi Karoubi, Ronen Segev, Mario F. Wullimann

**Affiliations:** ^1^Life Sciences Department and Zlotowski Center for Neuroscience, Ben-Gurion University of the NegevBeersheba, Israel; ^2^Graduate School of Systemic Neurosciences and Division of Neurobiology, Department Biology II, Ludwig-Maximilians-University of MunichMunich, Germany

**Keywords:** archerfish, brain atlas, choline acetyltransferase, dopamine, noradrenaline, tyrosine hydroxylase, visual system

## Abstract

Over recent years, the seven-spot archerfish (*Toxotes chatareus*) has emerged as a new model for studies in visual and behavioral neuroscience thanks to its unique hunting strategy. Its natural ability to spit at insects outside of water can be used in the laboratory for well controlled behavioral experiments where the fish is trained to aim at targets on a screen. The need for a documentation of the neuroanatomy of this animal became critical as more research groups use it as a model. Here we present an atlas of adult *T. chatareus* specimens caught in the wild in South East Asia. The atlas shows representative sections of the brain and specific structures revealed by a classic Nissl staining as well as corresponding schematic drawings. Additional immunostainings for catecholaminergic and cholinergic systems were conducted to corroborate the identification of certain nuclei and the data of a whole brain scanner is available online. We describe the general features of the archerfish brain as well as its specificities, especially for the visual system and compare the neuroanatomy of the archerfish with other teleosts. This atlas of the archerfish brain shows all levels of the neuraxis and intends to provide a solid basis for further neuroscientific research on *T. chatareus*, in particular electrophysiological studies.

## Introduction

Archerfish (*Toxotidae*) display a spectacular foraging behavior which can be studied under laboratory conditions accompanied by parallel electrophysiological investigations. Thus, it has emerged in the past years as a suitable model in neuroscientific research (Schuster, 2007; Mokeichev et al., 2010; Rischawy and Schuster, 2013; Ben-Tov et al., 2015). In the wild, the archerfish hunts in a very unique way: it spits a jet of water at insects that are generally sitting on leaves above the shallow waters where it lives (Timmermans, 2000; Schuster, 2007). To be efficient, the shot has to be very precise and the archerfish has developed this ability to an amazing level (Temple, 2007; Temple et al., 2010, 2013). It can be accurate up to two meters, adapts the strength of the jet to the distance and the size of the prey (Vailati et al., 2012) and uses a fast start strategy to be at the exact location where the bug will fall before it even touches the water (Wöhl and Schuster, 2006). This special hunting strategy can be used in laboratory (Gabay et al., 2013): archerfish can be trained to spit at targets on a screen (Vasserman et al., 2010; Ben-Simon et al., 2012; Ben-Tov et al., 2015), and hence offer a tool to track their responses to specific questions as faithfully as a mouse pulling a lever or a monkey pressing a key.

**FIGURE 1:**
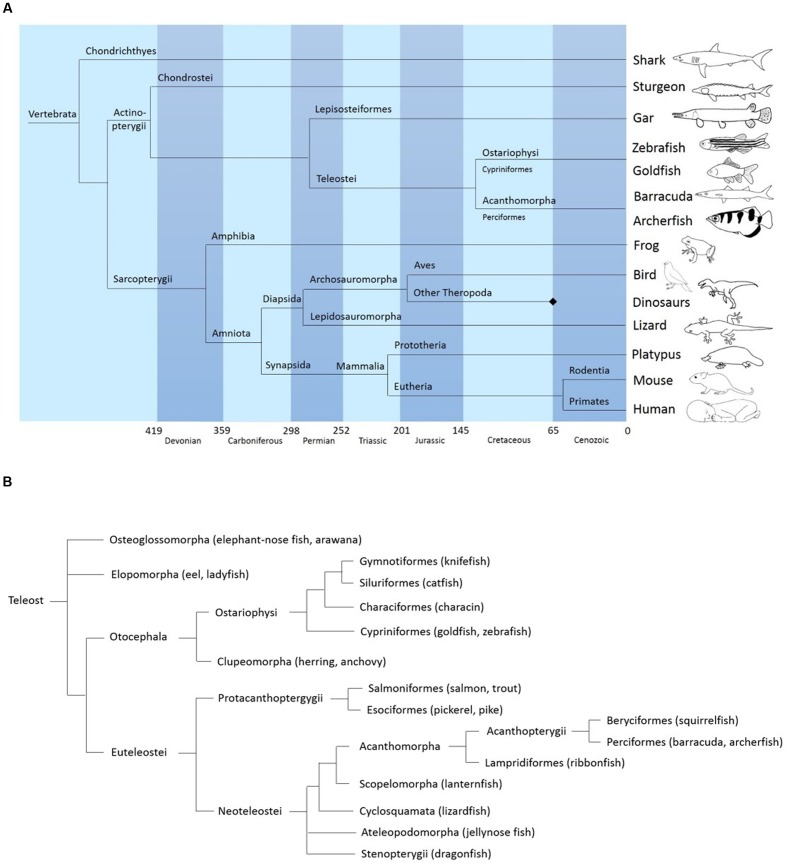
**(A)** Simplified phylogeny of vertebrates showing the position of acanthomorphs (including the archerfish) relative to other major vertebrate clades. Based on data from “The tree of life web project” (http://tolweb.org/tree/) (see also: Maddison et al., 2007) and *Vergleichende und funktionelle Anatomie der Wirbeltiere* Hildebrand and Goslow (2013). Springer-Verlag. **(B)** General phylogeny of teleosts showing the position of archerfish. Based on data from palaeos.com, “The tree of life project,” and Diogo et al. (2008) and Near et al. (2012).

Studying teleost brains has another appeal: because teleosts and mammals diverged in phylogeny long ago (**Figure 1A**) they can provide indications about structures and behaviors that might be conserved across the animal kingdom. It can be assumed with some confidence that a structure present both in a teleost and a mammalian brain will most likely be found in all vertebrates. In addition, in teleosts – being the larger taxon – higher variability among its members is observed, sometimes due to extremely specialized behavior in teleost fish. The archerfish for example shows high visual and motor specialization in its foraging behavior and for this reason is an interesting study object.

On the other hand, because of the early evolutionary divergence between teleosts and mammals, many structures evolved independently in the two groups and it is not surprising to observe a high diversity of mechanisms and brain organizations: evolution offers different approaches and solutions to a given problem. For example, since teleosts are able to perform tasks that require an isocortex for mammals such as recognition of a pop-out (Ben-Tov et al., 2013, 2015) or learn from peer’s observation (Schuster et al., 2006), we can assume that this recently evolved structure is not absolutely necessary for such complex behaviors.

In addition, teleosts being the largest taxon of the vertebrate line, high variability partly due to extreme specialization is observed among its members. The brain regions dedicated to those specialties will likely be hypertrophied compared to other teleosts lacking those abilities, rendering the investigation easier, in particular for electrophysiological investigations. The archerfish for example shows high vision and motor specialization in its foraging behavior and is therefore an interesting model for those research topics. However, a critical lack of neuroanatomical information exists in the study of the archerfish. A brain atlas is needed for relating studied behaviors to underlying neural circuits. Since several groups are working with archerfish, many of which conducting electrophysiology experiments, it is crucial to document the basic neuroanatomy of its major brain divisions, including description of possible specificities, in particular linked to its exceptional visuomotor abilities and higher integration processes.

The present study provides an atlas of the *Toxotes chatareus* brain. We identified the different structures mainly by comparison with other teleost fish and placing the archerfish in amore general phylogenetic context (**Figure 1B**). Specifically, in generating the archerfish brain atlas, we used the brain atlas of the zebrafish, *Danio rerio* (Wullimann et al., 1996), a prominent fish model animal representing a species within the ostariophysan clade that also include carps or catfish. In addition, because archerfishes, as percomorphs, belong to the acanthomorph clade we use in a complementary way the nomenclature of two other acanthomorphs: the cyprinodontiform fish *Nothobranchius furzeri* (D’Angelo, 2013) and the perciform *Dicentrarchus labrax* (Cerda-Reverter et al., 2001a,b, 2008). Since acanthomorphs show many neuroanatomical differences in comparison to ostariophysan fishes (Northcutt and Wullimann, 1988; Butler et al., 1991; Wullimann et al., 1996), in particular in the visually related structures, the nomenclature of the zebrafish only serves as a general neuroanatomical basis. We used additional staining revealing the catecholamine and cholinergic systems to confirm the identification of particular brain structures such as various dopaminergic and noradrenergic systems like the posterior tuberculum and the locus coeruleus (LC) respectively or all motor nuclei. Photomicrographs of Nissl-stained sections as well as corresponding schematic sketches are presented in this paper along with a description of the most characteristic brain structures.

## Materials and Methods

### Histochemical Nissl-Staining

All experiments were approved by the institutional animal care and use committee and in accordance with the laws of the State of Israel. Brains of archerfish were extracted from previously anesthetized and killed animals for further processing. Six adult archerfish, *Toxotes chatareus*, of size ranging from 5 to 8 cm were deeply anesthetized and euthanized with a 0.06% solution of ethyl 3-aminobenzoate methane sulfonate (MS 222; A.5040 Sigma). Each brain was removed from the skull and fixed in a solution of 4% paraformaldehyde for 72 h at 4°C. Afterward, brains used for Nissl-stains were washed in 70% ethanol overnight, progressively dehydrated up to 100% ethanol and then transferred into butanol before embedding in paraffin. The tissues were then cut into transverse sections of 7 μm with a rotary microtome. After removing of the paraffin and rehydration, the slices were stained with Cresyl Violet acetate (C-5042, Sigma) at 0.1% with a few drops of glacial acetic acid. They were finally rinsed in distilled water, dehydrated with ethanol, transferred into Xylene and coverslipped using Eukit as mounting medium.

### Immunohistochemical Staining

Archerfish brains (6) fixed for 48 h at 4°C in 4% paraformaldehyde were provided by the Segev lab (Israel) and further processed at LMU Munich at Martinsried (Germany). Cryoprotection of brains in 30% sucrose solution at 4°C took place overnight, and the brains were then embedded in TissueTek (tissue freezing medium, ordered from Leica, produced by Jung #14020108926) and cryosectioned (Leica, CM 3050 S) at 30 μm before thaw mounting onto Superfrost Plus glass slides (ordered from Carl Roth, produced by Thermo Scientific #H867.1).

Immunohistochemical incubations were done in a humid chamber. TissueTek was washed off the cryosections with PBT (phosphate buffered saline, PBS, pH 7.38, +0.1 % Tween 20) and blocked with blocking buffer (2% normal donkey serum, 0.5% Tween 20, 0.5% Triton X-100, 0.1% fish gelatine in PBS) for 1 h at room temperature before exposition to a primary antibody diluted in blocking buffer at 4°C for 1–3 days.

A mouse-anti tyrosine hydroxylase (TH) primary antibody (Millipore#MAB316) or a goat anti-choline acetyltransferase (ChAT) primary antibody (Millipore#AB144P) was used, both at a dilution of 1:200. After washing in PBT, the sections were incubated with the secondary antibody. Respective secondary antibodies used were donkey-anti-mouse-Alexa555 (Molecular Probes#A-31570 at 1:400 (TH) and either donkey-anti-goat-Cy3 (Dianova#705-165-147) at 1:300 or donkey-anti-goat-Alexa488 (Molecular Probes#A-11055) at 1:400 (ChAT) diluted in blocking solution for 3 h at room temperature. Finally, sections were washed in PBT and counterstained with DAPI (40-6-diamidino-2-phenylindole; Carl Roth #6335.1) used at 1 μg/ml working solution (1:1000 from stock solution: 1 mg/ml) and washed in PBS. Slides were then mounted with Vectashield (ordered from Enzo, produced by Vector #VC-H-1400-L010) and coverslipped. We have previously used the antibody against TH (Yamamoto et al., 2010, 2011) and ChAT (Mueller et al., 2004) doing various controls. The immunohistochemical results for the archerfish revealed very reliably only expected cells groups as seen in other teleost brains.

### Image Processing

A Leica microscope DM2500 equipped with a digital camera for bright field microscopic images was used to photograph the sections stained with cresyl violet. The images, sampled every 70–100 microns were then processed by means of GIMP2 (*GNU Image Manipulation Program*, free software) in order to correct for small differences in brightness and contrast, as well as to correct minor tissue artifacts. Photomicrographs of immunofluorescent sections were taken with a light/fluorescence microscope (Nikon Eclipse 80i; Nikon Instruments Inc.) equipped with Nikon Plan Fluor 10x/0.30 and a Nikon Digital Sight DS-U1 Photomicrographic Camera (Nikon Instruments Inc.) and LUCIA-G5 software. Raw pictures were eventually slightly adapted for contrast using Corel Photo Paint9 and final plates were mounted using Corel Draw 9 (Corel Corporation, Ottawa, ON, Canada).

### Neuroanatomical Analysis

The nomenclature is based on the one developed by Braford and Northcutt (1983) for the goldfish and Wullimann et al. (1996) for the zebrafish, and furthermore includes information from Cerda-Reverter et al. (2001a,b, 2008) and D’Angelo (2013) on *Dicentrarchus labrax* and *Nothobranchius furzeri*, respectively (see Introduction for justification).

## Results and Discussion

Among teleosts, the percomorphs form a sub-order of the acanthomorphs which represent the largest taxon of vertebrates with approximately 10000 species, amidst which we find the archerfish (**Figure 1**). Although one finds striking conservation of many structures within the order of teleosts, percomorph brain organization differs significantly from non-percomorph fish. In general, the pretectal organization of percomorphs is more complex: an intricate network of fibers and nuclei constitutes a major visual pathway leading from retinal ganglion cells to the hypothalamus, with various pretectal nuclei involved as relay centers (Sakamoto and Ito, 1982; Northcutt and Wullimann, 1988; Striedter and Northcutt, 1989; Butler et al., 1991; Shimizu et al., 1999; Yang et al., 2007). The most prominent of these nuclei is the large nucleus glomerulosus (NG) in the pretectum of acanthomorphs that is absent in other teleost taxa, but seems to be homologous to the posterior pretectal nucleus of basal teleosts, such as osteoglossomorphs (Butler et al., 1991; Wullimann et al., 1991).

Furthermore, in percomorphs, the inferior lobe of the hypothalamus is considered a multisensory integration center as it seems to be connected with several sensory systems. Although highly visually dominated fishes, the inferior lobe of percomorphs also receives gustatory afferents from the SGN, as for example in the sunfish *Lepomis cyanellus* (Wullimann, 1988) or the cichlid *Oreochromis niloticus* (Yoshimoto et al., 1998).

### Telencephalon

**FIGURE 2:**
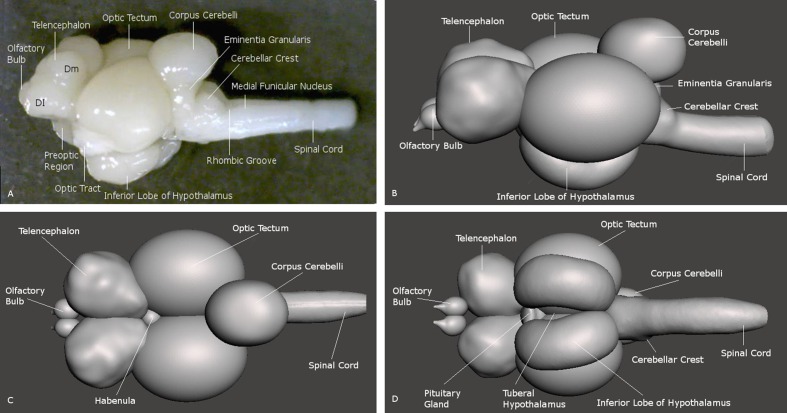
**Brain of the archerfish *Toxotes chatareus* shown in lateral view (A)** and in reconstructions seen from lateral **(B)**, dorsal **(C)**, and ventral **(D)**. Dm medial zone of dorsal telencephalic area (pallium), Dl lateral zone of dorsal telencephalic area (pallium).

The general anatomy of the telencephalon in the archerfish presents itself as expected from what is known in other acanthomorph teleosts, including percomorphs (Cerda-Reverter et al., 2001a; Pepels et al., 2002; Burmeister et al., 2009; D’Angelo, 2013). Positioned at the rostral end of the brain, the archerfish telencephalon exhibits two telencephalic hemispheres with various large pallial zones, more numerous than in zebrafish for example, and various ventral telencephalic nuclei. In addition, the olfactory bulbs are directly adjacent rostrally to the hemispheres and thus, do not show long secondary olfactory tracts (**Figure 2**).

**FIGURE 3:**
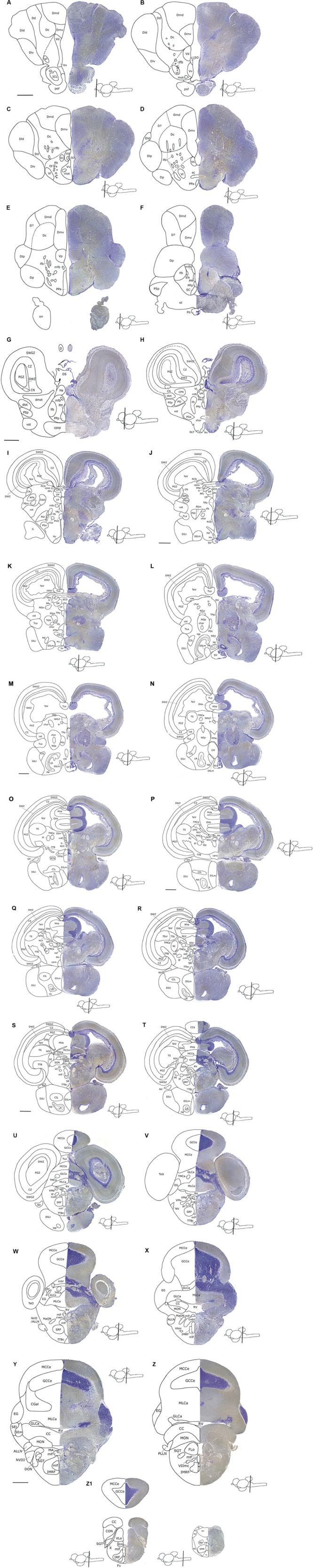
**An atlas of the brain of the archerfish *Toxotes chatareus*.** Nissl-stained (creysl violet) transverse sections (shown on the right) from telencephalic **(A–F)** via diencephalic **(G–L)**, mesencephalic **(G–W)** to rhombencephalic **(P–Z1)** levels with corresponding schematic drawings (shown on the left). Scale bar for all panels: 0,5 mm. See abbreviation list and text for details.

**FIGURE 4:**
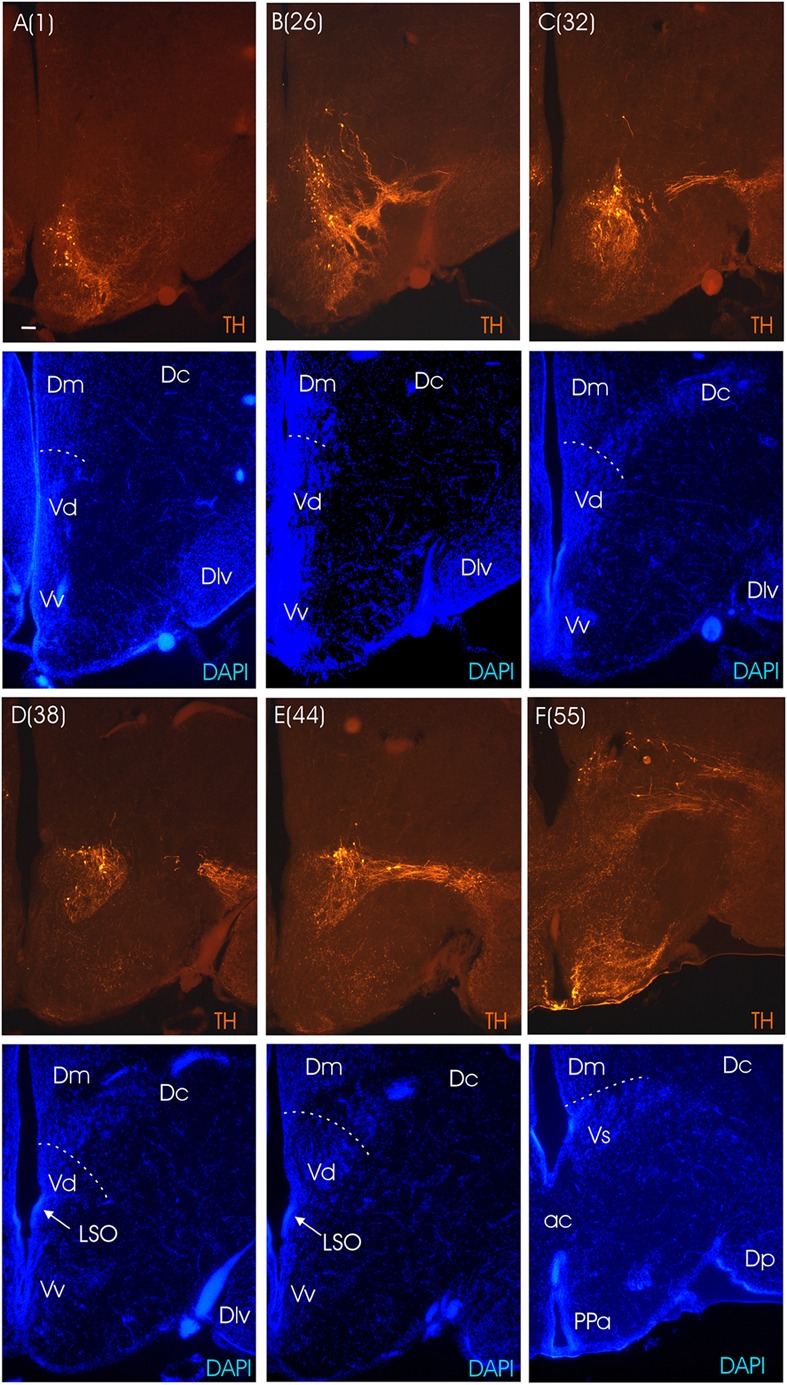
**Consecutive transverse sections of the archerfish telencephalon ranging from anterior to posterior **(A–F)**.** Sections are immunostained for tyrosine hydroxylase (TH) with accompanying nuclear (DAPI) counterstain. Stippled lines indicate pallial-subpallial boundary. Note that all dopaminergic cell bodies are within the subpallium. Arabic numerals in brackets indicate the relative distance of sections. Scale bar in **(A)**: 0.1 mm (applies to all panels). See abbreviation list and text for details.

The main divisions of the archerfish telencephalon are the ventral area (V; composed of nuclei) and the dorsal area (D; composed of zones). The dorsal telencephalon is homologous to the pallium in mammals and other vertebrates. The ventral telencephalon possesses dorsal (Vd), ventral (Vv), lateral (Vl), commissural (Vc), supracommissural (Vs) and postcommissural (Vp) nuclei (**Figures 3A–E**). We used an immunohistochemical assay for TH, the rate limiting enzyme for catecholamines, in order to confirm the pallial-subpallial boundary formed toward the pallium by these ventral telencephalic nuclei. The large telencephalic TH positive population in teleosts is restricted to the subpallium and consists of only dopaminergic, not noradrenergic cells (see Yamamoto et al., 2011 for discussion). In the archerfish, these subpallial dopamine cells form a long chain of cells starting at anterior levels in the ventral nucleus (Vv), then shift increasingly more dorsally at more caudal levels where they lie in the lateral part of the dorsal nucleus (Vd) and more caudally even in the supracommissural nucleus (Vs). As expected, no dopaminergic cells lie within the archerfish pallium, and the pallial-subpallial boundary (see stippled lines in **Figure 4**) is therefore clearly indicated in these stainings when used in addition to the DAPI counterstain.

The dorsal telencephalon is composed of medial (Dm), central (Dc), dorsal (Dd), lateral (Dl) and posterior (Dp) zones (**Figures 3A–F**). The Dl is anteriorly subdivided into a dorsal (Dld) and a ventral (Dlv) division and postcommissurally an additional subdivision of Dl appears, namely the posterior one (Dlp). Ventral to the latter we recognize the posterior zone (Dp), supposedly representing the olfactory pallium (but see below). Dc is characterized by various clusters of large cells. We recognize in the archerfish – in acccordance with the finding of Mueller et al. (2011) in the zebrafish – that these central parts of Dc extend toward the ventricular pallial zone. The medial pallial zone consists of two zones, that is a cell dense ventromedial subzone (DMv^1^) and a cell-sparse dorsomedial subzone (Dmd). Both central and lateral pallial zones have relatively dense cell distributions and therefore appear darkly stained. Between Dc and Dl (in the anterior telencephalon only) there is a conspicuous cell-sparse zone which we call dorsal pallial zone (Dd) as it is clearly separate from Dl. However, it might developmentally arise from the lateral zone. This dorsal zone (Dd) fades out caudally, and the central zone is then adjacent to the lateral zone. In the central part of the central zone, that is, where the pallium approaches the subpallium, many fibers congregate into tracts partially contributing to the anterior commissure (ac) but also to the emerging lfb. The most caudal pole of the pallial telencephalon lies dorsal to the preoptic region (**Figures 3F,G**).

Rostrally to the telencephalon properly speaking are the paired olfactory bulb lobes whose more caudal parts lie ventrally to the telencephalon. The glomerular layer (GL) with its less densely packed cells surrounding the cell-dense internal cellular layer (ICL) are both easily distinguishable (**Figure 3A**). At the periphery of the bulb are the incoming primary olfactory fibers (pof).

The basis for understanding the functional neuroanatomy of the pallium in teleosts is strongly built upon knowledge from studies in cyprinid fish on brain development, adult connectivity and neurotransmitter distribution. Fortuitously, many connectional and electrophysiological studies have been done in the goldfish or carp, two cyprinids, belonging to the same family as the zebrafish *Danio rerio*. The zebrafish is the main teleost model for developmental research (reviews: Wilson et al., 2002; Wullimann and Mueller, 2004; Chapouton et al., 2007; Baier and Scott, 2009; Portugues and Engert, 2009; Norton and Bally-Cuif, 2010; Roussigné et al., 2012; Hocking et al., 2013; Weber and Köster, 2013). By a twist of fate, the zebrafish – when seen within the broad variation observed in teleosts – exhibits a relatively simple pallium. Basically, undivided medial (Dm), lateral (Dl), central (Dc) and posterior zones (Dp) are recognized. Because pallial masses undergo a process of lateroventral eversion, there is a growing consensus that the medial zone (Dm) represents the pallial amygdala (or ventral pallium) and that the lateral zone (or at least its ventral subdivision) corresponds to the medial pallium or hippocampus in comparative terms (reviewed in Wullimann and Mueller, 2004; Wullimann, 2009; Mueller and Wullimann, 2016). The central pallial zone has recently been suggested not to be confined to a centrally located pallial division as previously believed, but to extend toward the dorsal surface of the pallium (Mueller et al., 2011). Thus, the central zone is a histogenetic unit with its own ventricular zone and may correspond to the dorsal pallium (i.e., isocortex). What has previously been described as a separate dorsal pallial zone (Dd) in cyprinids is newly interpreted by Mueller et al., as part of the lateral pallial zone. There is substantial connectional information in cyprinids to support these claims (Wullimann and Meyer, 1993; Northcutt, 2006; Yamamoto and Ito, 2008). Equally importantly, behavioral tests involving selective lesioning of goldfish pallial divisions revealed that Dm and Dl underlie functions, commonly associated with respectively pallial amygdalar (fear avoidance) and hippocampal (spatial map) functions in mammals (Portavella et al., 2002, 2003, 2004a,b; Rodrìguez et al., 2002a,b; Salas et al., 2003; Broglio et al., 2005). A consensus also exists on the identification of the posterior pallial zone (Dp) as the homolog of the olfactory cortex (Wullimann and Mueller, 2004; but see Nieuwenhuys, 2009).

Regarding the pallium of acanthomorph fishes, various suggestions regarding additional subdivisions have been made in different species (Cerda-Reverter et al., 2001a; Pepels et al., 2002; Burmeister et al., 2009; D’Angelo, 2013). However, since in acanthomorphs there is hardly any functional information available like that described for the goldfish above, many of the identifications remain to be confirmed. In any case, connectional and histochemical studies in both cyprinids (Northcutt, 2006) and acanthomorphs (Murakami et al., 1983) as well as in more basal salmonids (Castro et al., 2003; Folgueria et al., 2004) do agree in identifying Dm as homolog of the pallial amygdala and part of Dl as homolog of the hippocampus. The archerfish, typical for acanthomorphs, has internal subdivisions of the basic zones discussed above for the zebrafish pallium. However, we are aware of various problems of misidentification without embryological, histochemical, physiological and behavioral evidence when comparisons are made with the simpler pallium of cyprinids. The fact alone that various solutions of subdivisions of the basic pallial medial, lateral, dorsal and central zones have been suggested in acanthomorphs shows that there is inherent controversy in how to interpret these subdivisions. The potentially most controversial point in our description of the archerfish pallium is the delineation of Dc because we claim that its commonly accepted central, large-celled part has a stalk-like continuation toward the everted ventricle, at least in the anterior telencephalon. We indicate this stalk in the atlas plates as belonging to Dc (**Figures 3A–C**). In the caudal telencephalon, this stalk is not as obvious anymore and we indicate there preliminary boundaries for pallial zones (**Figures 3D–F**). In this respect, parvalbumin (Mueller et al., 2011) and calretinin (Castro et al., 2006) have been used in the zebrafish pallium to outline Dl/Dc versus Dm, respectively, and similar studies in archerfish with calcium-binding proteins may reveal more clearly the extent of pallial divisions such as Dc. In general, we remain conservative with numbers of pallial subdivisions in the archerfish. For example, olfactory bulb connections are not known in the archerfish. But these are crucial for identifying with certainty the posterior pallial zone (Dp). Fewer problems arise with the ventral, subpallial telencephalon and its nuclei, because the subpallium is more conservative in structure between all teleosts investigated.

### Diencephalon

The neuroanatomical terminology used is after Braford and Northcutt (1983) and Wullimann et al. (1996) as modified for acanthomorphs in Wullimann (1988), Wullimann and Northcutt (1988), Butler et al. (1991), Cerda-Reverter et al. (2001b), Pepels et al. (2002), and D’Angelo (2013). For the identification of different regions in the diencephalon we use again the TH immunostainings and these data will be mentioned as we go along. For the identification of TH positive structures we rely on Rink and Wullimann (2001) and Yamamoto et al. (2010, 2011).

**FIGURE 5:**
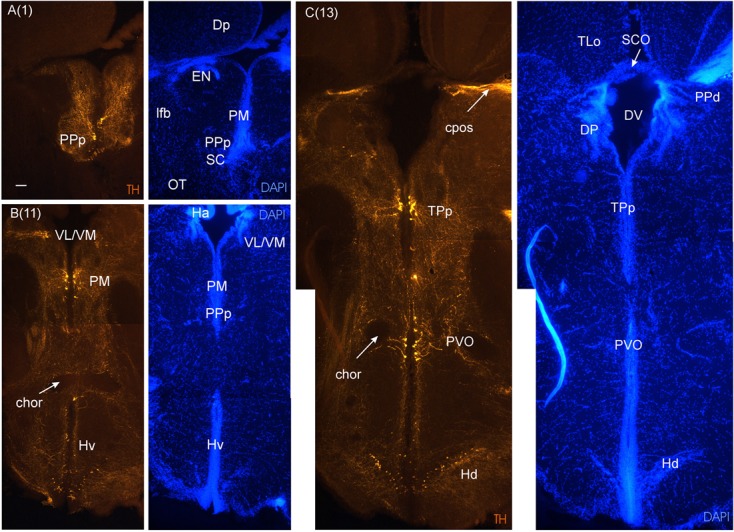
**Consecutive transverse sections of the archerfish diencephalon immunostained for tyrosine hydroxylase (TH) with accompanying nuclear (DAPI) counterstain.** Dopamine cells start out anteriorly in preoptic nuclei (PPp, PM seen in **A**), and are present more caudally in the ventral thalamus (VL/VM seen in **B**) and in the posterior tuberculum (TPp, PVO seen in **C**). In the hypothalamus, dopamine cells are present in the ventral and dorsal periventricular zones (Hv and Hd seen in **B and C**). Arabic numerals in brackets indicate the relative distance of sections. Scale bar in **(A)**: 0.1 mm (applies to all panels). See abbreviation list and text for details.

As we move caudally and reach the telencephalic commissural region, the optic nerves (on) appear ventrally to the telencephalon and form – after decussating in the optic chiasma - the optic tract at the junction between telencephalon and diencephalon (**Figures 3F,G**). Ventral to the anterior commissure, the preoptic area emerges (**Figure 3E**), with the anterior parvocellular preoptic nucleus (PPa). It contains numerous dopaminergic neurons (**Figure 4F**). More posteriorly, the PPa lies ventrally to the medial forebrain bundle (mfb). The lateral forebrain bundle (lfb) forms massive interconnections between telencephalon and diencephalon. At these preoptic levels, the magnocellular preoptic (PM), posterior parvocellular preoptic (PPp) and suprachiasmatic nuclei (SC) lie medially and ventrally to the lfb and – together with the EN – they are the only brain nuclei in this telencephalo-diencephalic stalk (**Figures 3F** and **5A**). The entopeduncular nucleus (EN) recognized in percomorphs is likely a misnomer. In zebrafish, there is a dorsal and ventral EN, with the ventral one corresponding to that seen in the archerfish. However, there is substantial evidence that this ventral EN in zebrafish corresponds to the bed nucleus of the stria medullaris (Mueller and Guo, 2009). Alternatively, it might be part of basal ganglia as suggested for zebrafish (Turner et al., 2016). At more caudal levels, where habenula (Ha) and postoptic commissure emerge (**Figure 3H**), this stalk still includes the PPp. The emergence of the ventral periventricular hypothalamus (Hv) is observed ventral to the horizontal commissure (chor). A conspicuous large-celled laterally lying nucleus is present at these levels in the archerfish hypothalamus which likely corresponds to the neuroendocrine lateral tuberal nucleus (NLT) seen in other teleosts where it expresses various neuropeptides (Cerda-Reverter et al., 2001b; Huesa et al., 2005; Castro et al., 2009).

Traditionally, the teleostean diencephalon has been considered to start anteriorly with the preoptic nuclei (PPa, PM, SC, PPp) and being followed caudally by the hypothalamus from which the optic nerve emerges. The PM includes the homolog of the mammalian paraventricular nucleus (Herget et al., 2014), the major neuropeptidergic nucleus involved in the hypothalamo-hypophysial axis. Since rather extensive, multipart magnocellular preoptic nuclei have been reported in salmonids (Saito et al., 2004) and acanthomorphs (Gómez-Segade and Anadón, 1986), appropriate neuropeptide staining (Herget et al., 2014) should be performed to clarify the extent of the PM in the archerfish. The PM also typically contains some dopamine neurons which we show here (**Figure 5B**). In mammals the paraventricular nucleus is considered hypothalamic. Thus, the two small preoptic mammalian nuclei would only correspond to part of the teleostean PPa. Furthermore, pretectum, dorsal and ventral thalamus (the latter is also called prethalamus) and epithalamus belong to the diencephalon. Boundaries toward the mesencephalon are sometimes hard to define and we discuss this as we go along. Thus, an interpretation of these facts in teleosts within the neuromeric model (Puelles and Rubenstein, 1993, 2003; mainly derived from data in amniote brains) is that all telencephalic and the so-called preoptic structures described above, plus all remaining hypothalamic divisions (described below) represent the secondary prosencephalon which is followed posteriorly by the prethalamic, thalamic and pretectal prosomeres.

**FIGURE 6:**
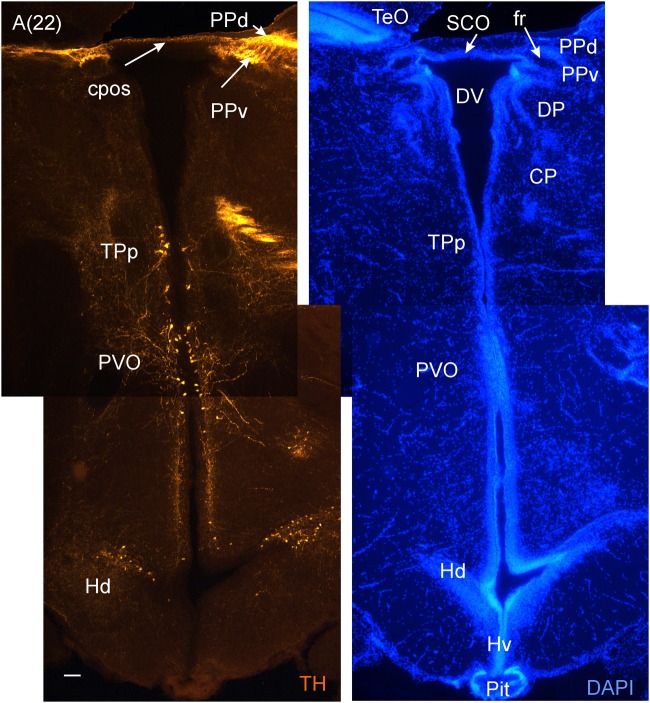
**A consecutive more caudal transverse section of the archerfish diencephalon **(A)** immunostained for tyrosine hydroxylase (TH) with accompanying nuclear (DAPI) counterstain showing pretectal (PPd/PPv), posterior tubercular (TPp, PVO) and hypothalamic (Hd) dopamine cells.** Arabic numerals in brackets indicate the relative distance of sections. Scale bar in (A): 0.1 mm. See abbreviation list and text for details.

**FIGURE 7:**
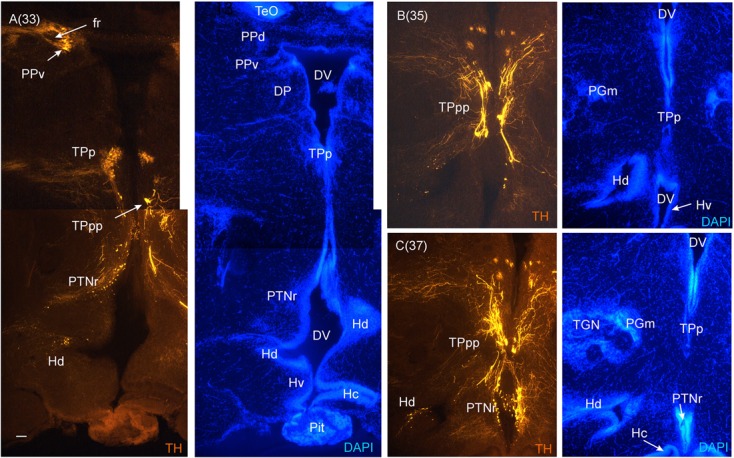
**Consecutive most caudal transverse sections of the archerfish diencephalon immunostained for tyrosine hydroxylase (TH) with accompanying nuclear (DAPI) counterstain.** At this caudal level, the periventricular posterior tubercular nucleus (TPp) exhibits in addition to small round cells, larger pear-shaped cells **(A–C)**. The pretectal (PPd/PPv) and dorsal hypothalamic (Hd) dopamine cells are still present and the posterior tuberal nucleus comes up (PTNr seen in **A–C**) which also exhibits dopamine cells. Note the absence of TH label in the caudal periventricular hypothalamic zone (Hc). However, see text for the likely expression of the *th2* gene whose enzyme product is not visualized by common antibodies against TH (Yamamoto et al., 2010, 2011). Arabic numerals in brackets indicate the relative distance of sections. Scale bar in **(A)**: 0.1 mm (applies to all panels). See abbreviation list and text for details.

At these anterior diencephalic levels the parvocellular superficial pretectal nucleus (PSp) can be seen to emerge, sandwiched between the most caudal telencephalon and preoptic stalk (**Figure 3F**), and it can be traced more posteriorly into the diencephalon, where it is replaced by other pretectal nuclei. The PSp is embedded superficially in the ot and has an important role in the visual pathway to the hypothalamus discussed above (Striedter and Northcutt, 1989). This explains its relatively large size and is not surprising for a visually dominated fish such as the archerfish. Also the ot is huge. After it has passed the optic chiasma it approaches the brain and divides up into dorsolateral and dorsomedial (dlot, dmot) as well as the ventrolateral optic tract (vot) because more pretectal nuclei emerge in its center. These include the magnocellular and intermediate superficial pretectal nuclei (PSm, PSi), as well as the central (CPN) and accessory pretectal (APN) nuclei (**Figures 3G–J**). Also closely associated with these nuclei is the dorsal accessory optic nucleus (DAO). More dorsomedially lie the dorsal and ventral periventricular pretectal nuclei (PPd/PPv; **Figures 3I–K**), closely associated with the posterior commissure (cpos) and retroflex fascicle (fr). Typically, PPd/PPv contain dopaminergic neurons (**Figures 6A** and **7A**). The subcommissural organ (SCO) forms the boundary toward the third ventricle ventral to the cpos. Another nucleus associated with the pretectum is the paracommissural nucleus (PCN) (**Figures 3I,J**). Many of these pretectal nuclei (PCN, PPd, CPN, DAO) project to the corpus cerebelli (CCe) via the anterior mesencephalo-cerebellar tract (TMCa; see below) in various teleosts, including the acanthomorph *Lepomis cyanellus* (Wullimann and Northcutt, 1988).

At rostrodorsal diencephalic levels one finds the epithalamus, with its major structures habenula (Ha), epiphysis (not shown in atlas sections), as well as the dorsal sac (DS). The two easily recognizable bulges of the Ha touch each other in the midline (**Figures 3G,H**).

Apart from pretectal nuclei already mentioned (PPd/PPv, PSp, PSi, PSm), there are additional visually related structures in the diencephalon. More caudal pretectal nuclei include the cortical nucleus (CN), the central (CPN) and accessory (APN) pretectal nuclei (**Figures 3H–J**). Ventrally to the Ha are the ventromedial and ventrolateral thalamic nucleus (VM/VL), and more posteriorly the dorsal thalamic nuclei, which are the anterior (not shown), the dorsal posterior and the central posterior nuclei (DP, CP). Typically, there are some dopaminergic cells in the ventral thalamus (**Figure 5B**) which correspond to the zona incerta in mammals.

In teleosts, PPd, PSp, VL, VM, CPN, CN receive direct retinofugal input, whereas DP and PSm receive tectal input. CP is an auditory related nucleus (see reviews Northcutt and Wullimann, 1988; Wullimann et al., 1996). Furthermore, the CN projects directly and the PSp via PSi to the nucleus glomerulosus (NG) (**Figures 3L–N**) which is positioned somewhat within and posterior to the preglomerular nuclei (see below). The NG in turn projects to the hypothalamic inferior lobe from where descending brain stem projection arise (review in Butler et al., 1991 and citations above). This is a major retino-pretecto-hypothalamo-bulbar visual pathway specific for acanthomorphs.

Ventrally to the pretectal and thalamic nucei lie the preglomerular nuclei, including the anterior, lateral, medial and caudal preglomerular nuclei (PGa, PGl, PGm, PGc; **Figures 3H–M**) and also the preglomerular tertiary gustatory nucleus (TGN). These nuclei constitute important relay centers for ascending sensory (for example gustatory and lateral line) projections to the pallium (Northcutt, 2006; Wullimann and Grothe, 2013).

In the medial diencephalon, ventral to dorsal and ventral thalamus, one finds the posterior tuberculum with its major nuclei, the periventricular posterior tubercular nucleus (TPp) and the paraventricular organ (PVO), both containing dopaminergic neurons (**Figures 5–7**). The TPp contains large pear-shaped dopamine neurons (TPpp) which are known to project to the ventral telencephalon in the zebrafish (Vd, arguably representing the striatum, see Rink and Wullimann, 2001). More caudally, the posterior tuberculum also contains the posterior tuberal nucleus (PTN), which divides into a rostral and caudal part in the archerfish. Only the rostral part has dopamine neurons (**Figure 7**). Alternatively, the PTNr might represent a second part of the PVO. In any case, in our preparations, the caudal PTN does not show TH positivity, but nevertheless highly likely contains TH/dopamine neurons because it may exclusively express the *th2* gene (see Discussion in next paragraph).

The periventricular hypothalamus is divided into dorsal (Hd), ventral (Hv) and caudal (Hc) zones (**Figures 3H–U**). The Hd is well defined because the lateral recess is in its center while the Hc has a posterior recess in its center. The Hv has no recess on its own. Both Hd and Hv contain dopaminergic cells in our preparations (**Figures 5–7**), but Hc does not. However, there are likely dopamine cells expressing the *th2* gene as its enzyme is not visualized with common TH antibodies (Yamamoto et al., 2010, 2011). The massive hypothalamic tissue lateral to the Hd forms the inferior lobe which contains various nuclei, that is, the medial and lateral diffuse nuclei (DILl, DILm), and the large-celled central nucleus (CIL). Also closely associated with the inferior lobe is the lateral torus (TLa). Ventral to midline (tuberal) hypothalamus, the lobular structure of the saccus vasculosus (SV) (**Figures 3J–N**) is easily recognizable (whereas the pituitary was lost in our preparations). The SV is a photoperiodic sensor which acts on seasonal gonadal changes in teleosts (Nakane et al., 2013) and is associated with a small nucleus in the posterior tuberculum, the NSV (**Figure 3L**), as previously shown in other species (Gómez-Segade and Anadón, 1988; Yáňez et al., 1997).

In the anterior part of the tegmental midbrain, the fibers of the horizontal commissure begin to form a tract which takes its path through the NG, exiting it anteroventrally, and finally crosses the midline at more anterior forebrain levels dorsal to Hv (**Figures 3H** and **5B**).

As we move toward more caudal diencephalic levels, the large caudal part of the unpaired posterior tuberal nucleus (PTNc; **Figure 3N**) with its star-like shape lies between the two glomerular nuclei and dorsal to the corpus mamillare (CM). Also at these caudal diencephalic levels, the nucleus of the medial longitudinal fascicle (NMLF) emerges (**Figures 3L–N**).

### Mesencephalon

The mesencephalic neuroanatomical terminology is after Wullimann et al. (1996) with modifications necessary for acanthomorphs made by Wullimann (1988), Wullimann and Northcutt (1988), Butler et al. (1991), Pepels et al. (2002), Cerda-Reverter et al. (2008), and D’Angelo (2013).

The most obvious structure of the archerfish midbrain is the optic tectum (TeO). As already discussed for diencephalic visual systems, the TeO is also well developed in this visually dominated fish species. The archerfish TeO emerges rostrally at the level of the preoptic stalk and fades out laterally to the CCe (**Figures 3G–W**). At the level of its greatest extent, four main layers can be clearly observed as in other teleosts (Meek, 1983). Centrally located around the tectal ventricle (TeV) is a loosely organized periventricular layer with very low cell density which is followed by the cell-dense periventricular gray zone (PGZ) in the narrower sense. Following Meek (1983), we indicate in the figures both of these sublayers as PGZ. Moving peripherally, a relatively thin deep white zone (DWZ) follows; it is bistratified with an inner gray zone and an outer layer appearing mesh-like. The DWZ is followed by the central white zone (CWZ; not further divided here). This zone has less dense layers of cells than the superficial white and gray zone (SWGZ) which is again peripherally adjacent. At the periphery of the SWGZ – often separated by an artificial gap – is the main optic input layer (Northcutt and Wullimann, 1988). It is – again with a gap – separated from the most peripheral marginal zone, which consists of the axons of the torus longitudinalis (TLo) (Wullimann, 1994). The TLo, which is only present in ray-finned fishes, runs along the entire rostrocaudal extent of the midline meeting point of the two tectal halves. Further caudally appears the second part of the alar midbrain, the torus semicircularis (TS; **Figures 3N–T**), which is covered by the enlarged tectal dome. In accordance with what is known in goldfish (Yamamoto et al., 2010), we recognize a central (likely auditory related) and a (likely lateral line related) ventrolateral subnucleus (**Figure 3R**) in the archerfish TS.

The anterior part of the basal midbrain (tegmentum) is characterized by vascular lacunae. In this general area, the fasciculus retroflexus (fr) is seen on its way from the habenula (Ha) to the interpeduncular nucleus (NIn). Also, centered in the mibrain tegmentum is the oculomotor nerve nucleus (IIIm) from which the third cranial nerve (NIII) takes its origin (**Figure 3O**). Caudal to the oculomotor nucleus is the trochlear motor nerve nucleus which is already in the hindbrain (see below). The ventral border of the midbrain tegmentum is represented by the ansulate commissure (cans) which is formed by the tectobulbar tracts (TTB) and indicates the floor of the midbrain (**Figure 3O**). At these levels, some major longitudinally running tracts are most evident. Most medially is the medial longitudinal fascicle (mlf) and more laterally is its counterpart, the lateral longitudinal fascicle (llf). While the mlf is an important premotor descending tract, the llf is the ascending tract of the lateral line and acoustic system. Also seen at these levels (**Figures 3Q,R**) is the anterior mesencephalo-cerebellar tract (TMCa; Wullimann and Northcutt, 1988) and the pretecto-isthmic tract (TPI) (Striedter and Northcutt, 1989). Caudal to the oculomotor nerve nucleus is the interpeduncular nucleus (NIn; **Figure 3P**) which is part of the hindbrain. The boundary of the midbrain tegmentum toward the hindbrain is otherwise hard to delineate. Likely the dorsal tegmental nucleus (DTN), the rostral tegmental nucleus (RTN), as well as the perilemniscal nucleus (PL) are part of the midbrain. The DTN in another acanthomorph, the Tilapia, is included in the nucleus lateralis valvulae (NLV) (Yang et al., 2004) which is discussed below. As usual in teleosts, there are no dopamine cells in the archerfish midbrain tegmentum. Generally, the midbrain basal plate structures form a far smaller part of the brain than the alar plate midbrain (Teo/TS).

### Rhombencephalon

The rhombencephalic neuroanatomical terminology is after Wullimann et al. (1996), with modifications necessary for acanthomorphs made by Wullimann (1988), Wullimann and Northcutt (1988), Butler et al. (1991), Pepels et al. (2002), Cerda-Reverter et al. (2008), and D’Angelo (2013).

The cerebellum is usually the most impressive structure in the teleostean rhombencephalon, only sometimes challenged in conspicuity by large vagal lobes, for example in carps and goldfishes. In the archerfish, the cerebellum includes a valvula (Va) (**Figures 3N–T**), which is only present in ray-finned fishes and extends into the tectal ventricle (TeV), a corpus cerebelli (CCe) (**Figures 3S–Z1**), and a vestibulolateral lobe, including the caudal cerebellar lobe (LCa; **Figures 3U–Z**) and the eminentia granularis (EG) (**Figures 3V–Z**). Cerebellar histology is uniform: granular and molecular layers intercepted by a ganglionic layer composed of Purkinje cells and eurydendroid cells. The latter correspond to the deep cerebellar nuclei of amniotes (Wullimann et al., 2011; Biechl et al., 2016). The CCe of the archerfish is comparable to the general vertebrate situation with the granular layer (GCCe) centrally and the molecular layer (MCCe) superficially. However, in the valvula, this order is reversed: there are two leaves with a peripheral granular (GVa) and an internal molecular layer (MVa). The caudal lobe has a large ventral molecular layer (MLCa) with a dorsally adjacent granular layer (GLCa) and an additional small granular layer at the dorsolateral edge of the rhombencephalic ventricle (RV). The EG extends lateroventrally to the granular layer of the CCe.

**FIGURE 8:**
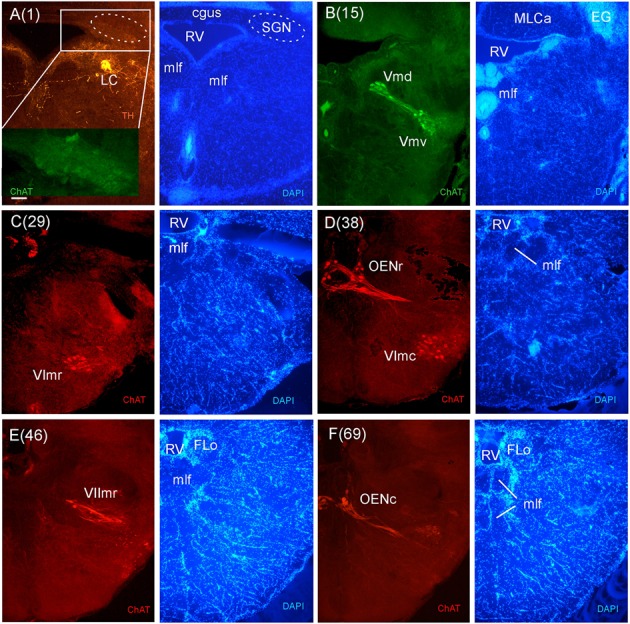
**Consecutive transverse sections of the archerfish anterior rhombencephalon double-immunostained for tyrosine hydroxylase (TH) and choline acetyltransferase (ChAT) with accompanying nuclear (DAPI) counterstain.** At anterior (isthmic) rhombencephalic levels, the noradrenergic locus coeruleus (LC) is present as is the secondary gustatory nucleus (SGN, seen in the same section in **A**). More caudally, the trigeminal (Vmd, Vmv), abducens (VIr, VIc), and rostral facial motor (VIImr) nuclei as well as the octavolateralis cholinergic motor neurons (OENr, OENc) are visualized **(B–F)**. Arabic numerals in brackets indicate the relative distance of sections. Scale bar in **(A)**: 0.1 mm (applies to all panels). See abbreviation list and text for details.

**FIGURE 9:**
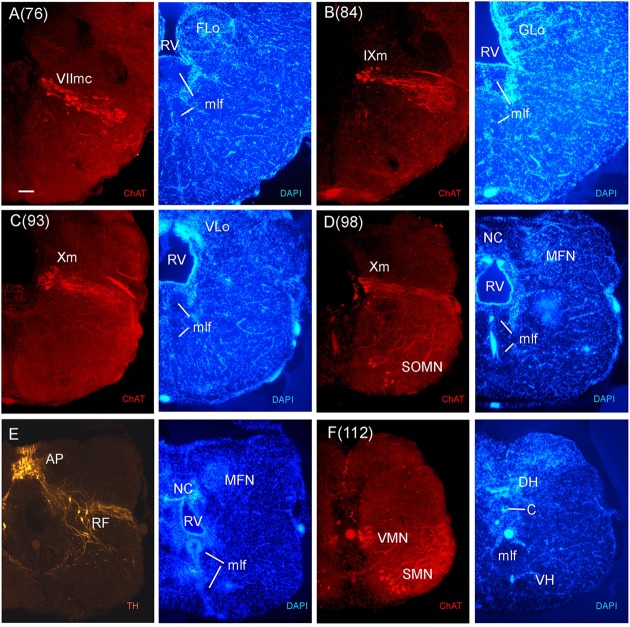
**Consecutive transverse sections of the archerfish posterior rhombencephalon double-immunostained for tyrosine hydroxylase (TH) and choline acetyltransferase (ChAT) with accompanying nuclear (DAPI) counterstain.** At these caudal rhombencephalic levels, the caudal facial (VIImc, seen in **A**), glossopharyngeal (IXm, seen in **B**), and vagal (Xm, seen in **C** and **D**) motor nerve nuclei are present, followed by the viscero- and somatomotor spinal cord neurons (SMN, seen in **F**). Between rhombencephalon and spinal cord there are some spino-occipital motorneurons (SOMN, seen in **D**). Note also the TH positive neurons of the area postrema (AP) and the reticular formation **(E)**. Arabic numerals in brackets indicate the relative distance of sections. Scale bar in **(A)**: 0.1 mm (applies to all panels). See abbreviation list and text for details.

For the analysis of the rhombencephalic brainstem, again our immunostains for catecholaminergic and cholinergic systems were highly useful. **Figures 8** and **9** represent a consecutive series of sections beginning at the level of the noradrenergic locus coeruleus (LC) (**Figure 8A**) down to the ventral motor neurons of the spinal cord (**Figure 9F**). This was done for two reasons: first, we could unambiguously identify some small but critical structures like the modulatory noradrenergic LC and the cholinergic secondary gustatory nucleus (SGN) (**Figure 8A**) or the exact location of the area postrema (AP) (**Figure 9E**). Secondly, we wanted to check whether there are any specializations in the motorneurons involved with the spitting behavior of the archerfish in comparison to other teleosts (Ekström, 1987; Pérez et al., 2000; Clemente et al., 2004; Mueller et al., 2004).

In stains for ChAT, we found in addition to the oculomotor (IIIm) and the trochlear (IVm) motor nerve nuclei (not shown in immunostains, but see **Figures 3O,P** of the Nissl-stain atlas), from rostral to caudal: the dorsal and ventral motor trigeminal nuclei, the two divisions of the abducens motor nerve nucleus, and the facial, glossopharyngeal and vagal motor nerve nuclei (**Figures 8** and **9**). While no motor nucleus was conspicuously enlarged, there appears to be a rostral facial motor nucleus in addition to a caudal one (**Figures 8E** and **9A**). Since the facial and the trigeminal motor nuclei innervate muscles associated with the mandibular and hyoid arch, respectively, which together are responsible for jaw movements, these nuclei are key to the spitting behavior. Teleost trigeminal and facial motor nuclei also innervate the dilator, adductor and levator muscles of the operculum, respectively (Gorlick, 1989). Also, we describe two populations of efferent octavolateralis cholinergic neurons (OENr/OENc; **Figures 8D,F**) and spino-occipital motor neurons (SOMN) (**Figure 9D**) in between the level of the vagal nerve motor nerve nucleus (Xm) and the spinal viscero- and somatomotor neurons (VMN/SMN; **Figure 9F**).

While cerebellum and motor nuclei provide vertebrate-typical landmarks within the rhombencephalon, there are various additional systems to be introduced as we proceed from the isthmic (anterior) rhombencephalon more caudally in the archerfish brain. The mlf and crossed tectobulbar tract (TTBc) continue down to most caudal brainstem and even spinal levels. In contrast, the llf is not present caudal to the medial octavolateralis nucleus (MON) because most of its ascending fibers originate from it. One can also follow the TMCa on its way into the cerebellum. It is joined by TMCp whose main origin is in the NLV (**Figures 3P–R**), a large nuclear mass at the base of the cerebellum (Wullimann and Northcutt, 1988). Various fiber bundles accompany the TMCs, but do not form a distinct anterior cerebellar tract (as seen for example in zebrafish). Also, various fiber systems enter the cerebellum from caudally, including the posterior cerebellar tract (PC). The nucleus isthmi (NI) (**Figure 3R**) with its ventrally emerging pretecto-isthmic tract (TPI) is another conspicuous isthmic structure. Also, the central gray (GC) starts out at isthmic levels and continues caudally. Slightly more caudally, the secondary gustatory nucleus (SGN) (**Figures 3S,T**) emerges with its typical commissure (cgus). Double-labeling of ChAT and TH show that the noradrenergic LC lies at the same level as the cholinergic SGN (**Figure 8A**), as is the pre-eminential nucleus (PE) and the superior raphe (SR) (**Figure 3T**). Also, the superior reticular formation (SRF) is present at these levels.

By convention, the SRF is present down to the level of the first branchiomeric (i.e., trigeminal) motor nerve nuclei which consist of a medial and lateral subnucleus (**Figures 3V** and **9A**), with the medial part extending far more rostrally. At levels of the sensory area of the medial octavolateralis nucleus (MON) (**Figures 3X–Z**) and the vestibular/auditory nuclei (such as the magnocellular, tangential, descending and anterior octaval nuclei; MaON, T, DON, AON), the SRF is followed by the intermediate reticular formation (IMRF). In cyprinids, only the AON and the auditory part of the DON have ascending projections to the diencephalon, whereas all octaval nuclei have descending projections (Wullimann and Grothe, 2013). Rostral and caudal abducens motor nerve nuclei are also present in this area (VImr, VImc; **Figures 3X** and **8C,D**). In this intermediate medullary area, an extensive system of ventral rhombencephalic commissures (cven) arises and continues caudally. Also, the octaval (VIII) and the anterior and posterior lateral line nerve roots (ALLN/PLLN) are seen at these levels (**Figures 3W–Z**). Among the more prominent tracts are the secondary gustatory tract (SGT) running from the facial (FLo) and vagal sensory lobes (VLo) toward the secondary gustatory nucleus (SGN). An unusual nuclear complex is also seen ventrolaterally to the EG: a lateral cell-dense and a medial cell-sparse subeminential nucleus (SEm, SEl; **Figure 3Y**). These have not been described to our knowledge in other acanthomorph species and their role needs further investigation.

The inferior reticular formation (IRF) follows more caudally in the general area of the chemosensory lobes, that are facial, glossopharyngeal and vagal lobes (FLo/GLo/VLo; **Figures 3Z–Z1**) which receive primary gustatory projections from respectively the facial, glossopharyngeal and vagal nerves, in teleosts (reviewed in Wullimann et al., 1996; Yoshimoto et al., 1998). In this caudal division of the archerfish medulla oblongata, we find the facial, glossopharyngeal and vagal motor nerve nuclei (**Figures 3Z–Z1**, **8** and **9**). Of note, two separate facial motor nuclei are present in the archerfish (**Figures 8E** and **9A**). The medial funicular nucleus (MFN) (**Figure 9E**) receives trigeminal projections, while the commissural nucleus of Cajal (NC) (**Figure 9E**) receives vagal interoceptive information (reviewed in Wullimann et al., 1996). The area postrema (AP) and the reticular formation in this posterior medullary region always contain dopaminergic and noradrenergic neurons (reviewed in Mueller and Wullimann, 2016) and they are also found in the archerfish (**Figure 9E**).

## Ethics Statement

All animal experiments were approved by the Institutional Animal Care and Use Committee of Ben Gurion University in the Negev and in accordance with the laws of the State of Israel. Brains of archerfish were extracted from previously anesthetized and killed animals for further processing.

## Author Contributions

All authors listed have made substantial, direct and intellectual contribution to the work and approved it for publication The data were generated by NK (Nissl atlas) and MW (immunostainings). NK, RS, and MW wrote the article.

## Conflict of Interest Statement

The authors declare that the research was conducted in the absence of any commercial or financial relationships that could be construed as a potential conflict of interest.

## Footnotes

1 A recent report on GABA cell patterns in another acanthomorph species interprets this area DMv as a subpallial, ventral telencephalic supracommissural nucleus (Vs-m) (see Maruska et al., 2016).

## Abbreviations

ac: anterior commissure
AP: area postrema
APN: accessory pretectal nucleus
C: central canal
cans: commissura ansulata
CC: crista cerebellaris
CCe: corpus cerebelli
ccer: cerebellar commissure
CGaL: common ganglionic layer of LCa and CCe
chab: habenular commissure
cgus: secondary gustatory commissure
chor: horizontal commissure
CIL: central nucleus of hypothalamic inferior lobe
CM: corpus mamilare
CN: cortical nucleus
CON: caudal octavolateralis nucleus
CP: central posterior thalamic nucleus
CPN: central pretectal nucleus
cpop: postoptic commissure
cpos: posterior commissure
cven: rhombencephalic ventral commissure
CWZ: central white zone of optic tectum
DAO: dorsal accessory optic nucleus
Dc: central zone of dorsal telencephalon
Dd: dorsal zone of dorsal telencephalon
DH: dorsal horn
DILl: diffuse nucleus of hypothalamic inferior lobe, lateral part
DILm: diffuse nucleus of hypothalamic inferior lobe, medial part
Dl: lateral zone of dorsal telencephalon
Dld: dorsal division of Dl
Dlp: posterior division of Dl
dlot: dorsolateral optic tract
Dlv: ventral division of Dl
Dm: medial zone of dorsal telencephalon
Dmd: dorsal division of Dm
dmot: dorsomedial optic tract
Dmv: ventral division of Dm
DON: descending octaval nucleus
Dp: posterior zone of dorsal telencephalon
DP: dorsal posterior thalamic nucleus
DS: dorsal sac
DTN: dorsal tegmental nucleus
DV: diencephalic ventricle
DWZ: deep white zone of optic tectum
EG: eminentia granularis
EN: entopeduncular nucleus
EONc: caudal efferent octavolateralis neurons
EONr: rostral efferent octavolateralis neurons
FLo: facial lobe
fd: funiculus dorsalis
fld: funiculus lateralis, pars dorsalis
flv: funiculus lateralis, pars ventralis
fr: fasciculus retroflexus
fv: funiculus ventralis
GC: griseum centrale
GCCe: granular layer of CCe
GL: glomerular layer (olfactory bulb)
GLCa: granular layer of LCa
GLo: glossopharyngeal lobe
GVa: granular layer of Va
H: hypothalamus
Ha: habenula
Hc: caudal zone of periventricular hypothalamus
Hd: dorsal zone of periventricular hypothalamus
Hv: ventral zone of periventricular hypothalamus
ICL: internal cellular layer (olfactory bulb)
IL: inferior lobe of hypothalamus
IMRF: intermediate reticular formation
IRF: inferior reticular formation
LC: locus coeruleus
LCa: lobus caudalis cerebelli
lfb: lateral forebrain bundle
llf: lateral longitudinal fascicle
LR: lateral recess of diencephalic ventricle
LSO: lateral septal organ
MA: Mauthner axon
MaON: magnocellular octaval nucleus
MCCe: molecular layer of CCe
mfb: medial forebrain bundle
MFN: medial funicular nucleus
MLCa: molecular layer of LCa
mlf: medial longitudinal fascicle
MON: medial octavolateralis nucleus
mot: medial olfactory tract
mfb: medial forebrain bundle
MVa: molecular layer of Va
MZ: marginal zone of optic tectum
NC: nucleus commissuralis of Cajal
NGa: nucleus glomerulosus (anterior part)
NGp: nucleus glomerulosus (posterior main part)
NI: nucleus isthmi
NInd: dorsal part of nucleus interpeduncularis
NInv: ventral part of nucleus interpeduncularis
NLT: nucleus lateralis tuberis
NLV: nucleus lateralis valvulae
NR: red nucleus (nucleus ruber)
NSV: nucleus of the saccus vasculosus
on: optic nerve
ot: optic tract
PC: posterior cerebellar tract
PCN: paracommissural nucleus
PE: pre-eminential nucleus
PGa: anterior preglomerular nucleus
PGc: caudal preglomerular nucleus
PGl: lateral preglomerular nucleus
PGm: medial preglomerular nucleus
PGZ: periventricular gray zone of the optic tectum
Pit: pituitary gland
PL: perilemniscal nucleus
PM: magnocellular preoptic nucleus
pof: primary olfactory fibers (olfactory bulb)
PPa: anterior parvocellular preoptic nucleus
PPd: dorsal periventricular pretectal nucleus
PPp: posterior parvocellular preoptic nucleus
PPv: ventral periventricular pretectal nucleus
PR: posterior recess of diencephalic ventricle
PSi: intermediate superficial pretectal nucleus
PSm: magnocellular superficial pretectal nucleus
PSp: parvocellular superficial pretectal nucleus
PT: posterior thalamic nucleus
PTNc: posterior tuberal nucleus, caudal part
PTNr: posterior tuberal nucleus, rostral part
PVO: paraventricular organ
RF: reticular formation
RTN: rostral tegmental nucleus (of Grover and Sharma, 1981)
RV: rhombencephalic ventricle
SC: suprachiasmatic nucleus
SCO: subcommissural organ
SEl: subeminential nucleus (lateral part)
SEm: subeminential nucleus (medial part)
SGN: secondary gustatory nucleus
SGT: secondary gustatory tract
SMN: (ventral horn) somatomotor neurons
SOMN: spino-occipital motor neurons
SR: superior raphe
SRF: superior reticular formation
SV: saccus vasculosus
SWGZ: superior white and gray zone of optic tectum
T: tangential octaval nucleus
tbs: tractus bulbo-spinalis
TeO: optic tectum
TeV: tectal ventricle
TGN (preglomerular): tertiary gustatory nucleus
TLa: torus lateralis
TLo: torus longitudinalis
TMCa: anterior mesencephalo-cerebellar tract
TMCp: posterior mesencephalo-cerebellar tract
TPI: pretecto-isthmic tract
TPp: periventricular nucleus of the posterior tuberculum
TPpp: pear-shaped cells of TPp (type 2 cells of Rink and Wullimann, 2001)
TSc: torus semicircularis, central nucleus
TSvl: torus semicircularis, ventrolateral nucleus
TTB: tractus tectobulbaris
TTBc: tractus tectobulbaris cruciatus
Va: valvula cerebelli
VAO: ventral accessory optic nucleus
Vas: vascular lacuna of midbrain tegmentum
Vc: central nucleus of ventral telencephalon
Vd: dorsal nucleus of ventral telencephalon
Vl: lateral nucleus of ventral telencephalon
VL: ventrolateral thalamic nucleus
VLo: vagal lobe
VM: ventromedial thalamic nucleus
VMN: (intermediate horn) visceromotor neurons
vot: ventrolateral optic tract
Vp: postcommissural nucleus of ventral telencephalon
Vs: supracommissural nucleus of ventral telencephalon
Vv: ventral nucleus of ventral telencephalon
DIV: **Cranial nerve structures:**
ALLN: anterior lateral line nerve
DIV: trochlear nerve decussation
GV: trigeminal ganglion
NI: olfactory nerve
NII: optic nerve
NIII: oculomotor nerve
NIV: trochlear nerve
NV: trigeminal nerve
NVI: abducens nerve
NVII: facial nerve
NVIII: octaval nerve
NIX: glossopharnygeal nerve
NX: vagal nerve
PLLN: posterior lateral line nerve
IIIm: oculomotor nerve nucleus
IIImr: oculomotor nerve root
IVm: trochlear motor nerve nucleus
Vmd: dorsal trigeminal motor nerve nucleus
VMv: ventral trigeminal motor nerve nucleus
VIc: caudal abducens motor nerve nucleus
VIr: rostral abducens motor nerve nucleus
VIIr: rostral facial motor nerve nucleus
VIIc: caudal facial motor nerve nucleus
IXm: glossopharyngeal motor nerve nucleus
Xm: vagal motor nerve nucleus
